# Longitudinal Screening for Oral High-Risk Non-HPV16 and Non-HPV18 Strains of Human Papillomavirus Reveals Increasing Prevalence among Adult and Pediatric Biorepository Samples: A Pilot Study

**DOI:** 10.3390/vaccines12080895

**Published:** 2024-08-08

**Authors:** Jordan Jacobs, Eugene Chon, Karl Kingsley

**Affiliations:** 1 Department of Clinical Sciences, School of Dental Medicine, University of Nevada-Las Vegas, 1700 W. Charleston Boulevard, Las Vegas, NV 89106, USA; 2 Department of Biomedical Sciences, School of Dental Medicine, University of Nevada-Las Vegas, 1001 Shadow Lane, Las Vegas, NV 89106, USA

**Keywords:** clinical sampling, human papillomavirus (HPV), biorepository saliva screening, high-risk oral HPV, nine-valent HPV vaccine

## Abstract

Most high-risk oral human papillomavirus research has focused on prevalent HPV16 and HPV18, with fewer studies focused on other high-risk strains incorporated into the nine-valent HPV vaccine. Therefore, the objective of this study was to determine the oral prevalence of non-HPV16 and non-HPV18 high-risk strains. A total of *n* = 251 existing biorepository saliva samples were screened using validated primers and qPCR. A total of *n* = 72 samples tested positive for HPV, including HPV31, HPV33, HPV35, HPV52, and HPV58. In addition, there were also significant increases in the prevalence of these high-risk strains (2011–2014, 21.3%) following the nine-valent HPV vaccine’s introduction (2015–2019, 36.2%). However, the distribution of HPV-positive samples was nearly equal among males and females (52.8%, 47.2%, respectively, *p* = 0.5485), although the majority (66.7%) of the HPV-positive samples were within the HPV vaccination age (11 to 26 years) or catch-up range (27 to 45 years). These data demonstrated that the prevalence of high-risk oral HPV may be higher than anticipated, highly concentrated among patients within the recommended vaccination age range, and may be increasing over time—providing new evidence and support for the nine-valent HPV vaccine that covers these additional high-risk HPV strains.

## 1. Introduction

High-risk oncogenic strains of human papillomavirus (HPV) have been demonstrated to be involved with many oral and head and neck tumors [1,2]. Recent evidence has demonstrated that high-risk HPV strains are capable of mediating and modulating the progression of oropharyngeal and other types of head and neck cancers, thereby influencing patient outcomes and reducing patient survival [3,4]. Moreover, many of these high-risk HPV strains have also been associated with the initiation and development of oral and other head and neck cancers, which presents many potential opportunities for prevention [5,6].

The vast majority of the research into the prevalence of high-risk oral strains has focused on HPV16 and HPV18, which are known to comprise the largest proportion of known infections to date [7,8]. These high-risk strains may also be associated with the vast majority of cervical cancers and other HPV-related diseases, exerting their effects through HPV early proteins E6 and E7 that downregulate tumor suppressors Retinoblastoma (Rb) and TP53 [9,10]. However, a growing number of studies now demonstrate that additional non-HPV16 and non-HPV18 high-risk strains that were not originally included in the initial studies of oral and head and neck cancers may not only be present but also functional in the initiation, development, and progression of these tumors [11,12].

Although HPV16 and HPV18 are very important high-risk HPV strains, many additional strains including HPV31, HPV33, HPV45, HPV52, and HPV58 are also associated with many types of cancer development and progression in a variety of tissues and organs, including oropharyngeal and head and neck cancers [13,14]. The recognition that the prevention of infection by these additional high-risk strains may significantly reduce morbidity and mortality has led to the incorporation of these additional high-risk strains into the modified nine-valent HPV vaccine, covering high-risk HPV16, HPV18, HPV31, HPV33, HPV45, HPV52, and HPV58, as well as the low-risk strains HPV6 and HPV11 associated with the majority of anogenital warts [15,16,17]. Although these additional high-risk strains are well studied among other cancers, fewer studies have focused on these high-risk strains among oral or head and neck cancers [18,19,20].

In addition, most of the research in these areas has been focused on obtaining the one-time, cross-sectional prevalence of high-risk HPV16 and HPV18, although some researchers have started to perform longitudinal studies to track changes in prevalence over time [21,22,23]. These studies have demonstrated increases in both the prevalence and incidence of HPV16 and HPV18 over time, although much less is known about high-risk non-HPV16 and non-HPV18 strains [24,25,26]. For example, although a recent systematic review and meta-analysis of oral HPV strains confirmed the high prevalence of HPV16 and HPV18 among oropharyngeal cancer patients, HPV33 and HPV58 (included in the nine-valent HPV vaccine), as well as the additional strain HPV35, were also among the most prevalent in the oral cavity [27]. This report of high oral HPV35 prevalence has been demonstrated in numerous other screening studies of oropharyngeal cancer, which suggest that this strain may be much more common among oral cancer patients than the high-risk cervical cancer-associated strain HPV45 that is covered by the nine-valent vaccine [20,28,29,30]. Some evidence now suggests that the oral prevalence of HPV35 may not only be high but may also be increasing over time, suggesting that the screening of this strain may be of particular relevance for screening studies of high-risk oral HPV strains [31,32,33]. In fact, despite the high prevalence of the high-risk strain HPV45 in cervical cancers, the few studies screening for this strain have revealed a strikingly low prevalence among oral and oropharyngeal cancers [34,35].

Due to the recent nature of these discoveries and the limited information available regarding non-HPV16 and non-HPV18 high-risk HPV strains, the primary goal within the current study involved the analysis and screening of saliva samples within an existing biorepository to reveal a more complete understanding of oral HPV infections, as well as any temporal trends or associations over time.

## 2. Materials and Methods

### 2.1. Study Approval

This study involved a retrospective analysis of previously collected samples stored within a biorepository. As a retrospective study with no clinical sample collection or patient interactions, this study was reviewed and approved by the Institutional Review Board (IRB) as Research Exempt for studies that do not require informed consent according to the United States (US) Department of Health and Human Services (HHS) code of federal regulations (CFR) 45 CFR 46, which states that research using previously collected data or specimens that cannot be linked with patient-identifying information or personal private information. The protocol titled “Retrospective analysis of microbial prevalence from DNA isolated from saliva samples originally obtained from the University of Nevada, Las Vegas (UNLV) School of Dental Medicine (SDM) pediatric and clinical population” was filed and approved by the UNLV Office for the Protection of Research Subjects (OPRS) and IRB under Protocol 1717625-1.

### 2.2. Biorepository Sample Collection

The biorepository consisted of clinical saliva samples derived under the original sample collection Protocol 1305-4466M titled “The Prevalence of Oral Microbes in Saliva from the University of Nevada, Las Vegas School of Dental Medicine (UNLV-SDM) Pediatric Adult Clinical Population”. The original study inclusion criteria required that samples be collected from UNLV-SDM patients of record and that participation be strictly voluntary. Exclusion criteria included any patients that did not wish to participate. Briefly, unstimulated saliva was collected using sterile collection tubes and stored for processing at −80 °C. Labels with randomly generated identifiers were used to prevent any personal or private patient information from being associated with any particular sample. Only basic demographic information was noted, such as patient age, sex, and race or ethnicity. No chart numbers, patient birth dates, or other specific information was collected or available to the study authors. Samples were collected on randomly selected days on alternating months over the time period between 2011 and 2019.

### 2.3. DNA Isolation

DNA was previously isolated from all saliva samples within the biorepository using the TRIzol (phenol–chloroform) extraction procedure from Invitrogen (Waltham, MA, USA), as detailed in previous studies [19,20]. The quantification and assessment of the sample-derived DNA were performed using a NanoDrop 2000 spectrophotometer obtained from Fisher Scientific (Fair Lawn, NJ, USA). The quantity and quality of sample-derived DNA were evaluated utilizing the A260 and A280 nm absorbance readings and ratio, as outlined previously [19,20].

### 2.4. qPCR Screening

This study involved *n* = 251 DNA biorepository samples isolated from patient saliva. Screening for high-risk non-HPV16 and non-HPV18 was performed using validated primers for each specific HPV strain (HPV31, −33, −35, −52, −58). The screening protocol involved the use of the SYBR Green Universal qPCR Master Mix obtained from Applied Biosystems (Waltham, MA, USA) and the manufacturer’s recommended protocol, as previously described [19,20]. Previously validated primer sequences were obtained from Eurofins Scientific (Louisville, KY, USA), as outlined in Table 1 below [19,20]:

### 2.5. Statistical Analysis

Data regarding the demographic characteristics of the study sample were compiled for analysis using Chi Square, the recommended method for statistical comparisons of non-parametric, categorical data. In addition, the qPCR results were also categorized (HPV-positive, HPV-negative) and analyzed using Chi Square statistics. Descriptive statistics and Pearson’s correlations (R) were generated using Microsoft Excel 2021, Office 365 Version (Redmond, WA, USA), and Chi Square analysis was performed using GraphPad Prism software, Version 8 (San Diego, CA, USA), as previously described [19,20,36].

## 3. Results

### 3.1. Demographic Analysis of Study Sample

This study screened *n* = 251 biorepository samples (Table 2). An analysis of the demographics demonstrated that these samples were nearly equal with respect to the proportion of males (50.6%) and females (49.4%) which closely matched the overall proportion within the clinical population, *p* = 0.8414. Most samples had some demographic characteristics regarding race or ethnicity (95.6% or *n* = 240/251). This analysis revealed that most samples were derived from non-White or minority patients (74.2%), similar to the percentage within the overall patient clinic population (70.6%), *p* = 0.5085. More detailed analysis revealed that these samples were derived from minority patients that self-identified as Hispanic or Latino (30.4%), Black or African American (20.4%), and Asian or Pacific Islander (20.0%). In addition, the average age for all study samples was 25.2 years, which was similar to the overall clinical population of 26.4 years, *p* = 0.1806.

### 3.2. High-Risk Oral HPV Screening Results

To determine if the samples identified met the standards for qPCR screening, an analysis of DNA quality and quantity was performed (Table 3). These data demonstrated that the average DNA concentration for pediatric samples was 312.4 ng/uL with a range of 221 to 452 ng/uL, which was well above the recommended minimum concentration of 10 ng/uL for optimal qPCR screening. Adult samples had a higher average DNA concentration of 431.2 ng/uL with a range of 347 to 558 ng/uL, which also met the minimum recommended DNA concentration standard for qPCR processing. The purity of pediatric and adult samples was calculated using the ratio of absorbance readings measured at absorbances of A260 and A280 nm. These data demonstrated that the quality of DNA from pediatric (A260:A280 ratio of 1.76) and adult (A260:A280 ratio of 1.79) samples met the minimum qPCR standard of an A260:A280 ratio of 1.70.

**Figure 1:**
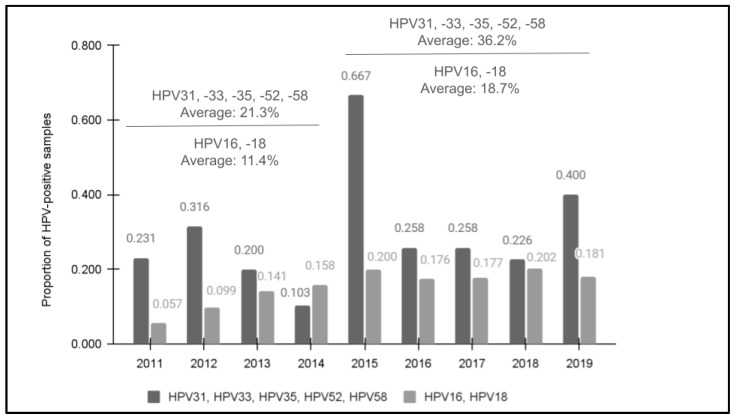
The results of qPCR screening for HPV31, HPV33, HPV35, HPV52, and HPV58. Screening demonstrated that 28.7% (*n* = 72/251) were HPV-positive for one or more of the high-risk strains with an increase observed between 2011 (23.1%) and 2019 (40.0%). The overall average of 2011 to 2014 (21.3%) was significantly lower than following the nine-valent vaccine introduction from 2015 to 2019 (36.2%), *p* = 0.019. In addition, previous data from this clinic population for HPV16 and HPV18 data also revealed increasing trends between 2011 (5.7%) and 2019 (18.1%).

All samples met the minimum standard for qPCR quantity and were then confirmed as having human DNA using the positive control primer for glyceraldehyde 3-phosphate dehydrogenase (GAPDH) prior to screening for high-risk HPV using qPCR (Figure 1). The screening results demonstrated that 28.7% (*n* = 72/251) of samples were found to harbor at least one of these high-risk HPV strains. In addition, an increasing trend in prevalence was observed between 2011 (23.1%) and 2019 (40.0%). Although there was one year with an unusually high number of HPV-positive samples (2015), the overall average of the four years 2011 to 2014 prior to the introduction of the nine-valent vaccine (21.3%) was significantly lower than the average of the five years following the modified HPV vaccine rollout and introduction from 2015 to 2019 (36.2%), *p* = 0.019. This revealed a moderate, positive correlation between the year the sample was taken and the potential for HPV positivity, R = 0.384.

Comparative data from a previous study of this clinic population revealed that HPV positivity for HPV16 and HPV18 data also demonstrated an increasing trend in prevalence between 2011 (5.7%) and 2019 (18.1%) that also had an increased average for the latter five years following the nine-valent HPV vaccine’s introduction from 2015 to 2019 (18.7%) compared with the first four years of the analysis from 2011 to 2014 (11.4%) [27]. Overall, a higher percentage of samples tested positive for one of the five high-risk HPV strains in each year compared with the percentage of samples previously testing positive for either HPV16 or HPV18.

A more detailed analysis of the HPV-positive and HPV-negative results was performed (Table 4). This analysis revealed that no significant differences were found between females and males in the HPV-positive (47.2% and 52.8%, respectively) or HPV-negative (50.3% and 49.7%, respectively) categories, *p* = 0.5485. Moreover, the percentage of samples from White and minority patients was also similar between the HPV-positive (20.8% and 72.3%, respectively) and HPV-negative (26.3% and 70.4%, respectively) groups, *p* = 0.1951. The proportion of HPV-positive and HPV-negative minority subgroups was also similar, such as the percentages of Hispanics or Latinos (29.2% and 29.1%, respectively), Black or African Americans (20.8% and 18.9%, respectively), and Asian or Pacific Islanders (18.1% and 19.6%, respectively). Furthermore, two-thirds (66.7%) of the HPV-positive samples were within the HPV vaccination age (11 to 26 years) or catch-up range (27 to 45 years), which was similar to the percentage of HPV-negative samples within the same age ranges (64.8%), *p* = 0.4443.

### 3.3. Comparison of HPV Screening Results with Recommended Vaccination Age

**Figure 2:**
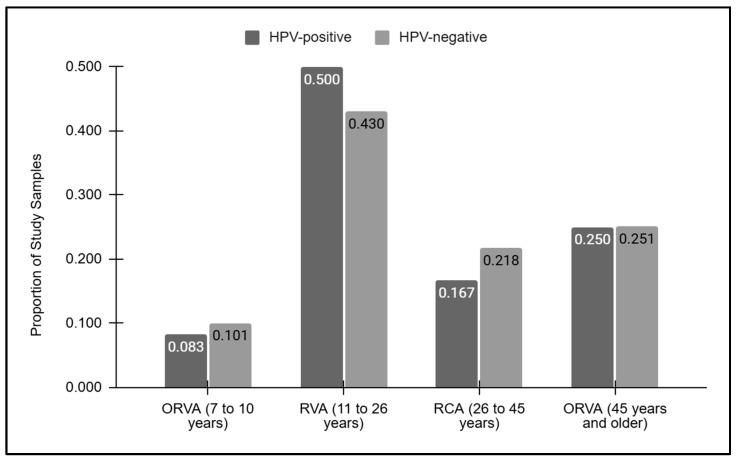
Vaccination grouped ages for the HPV-positive and HPV-negative samples. Samples below or outside the recommended vaccination age (ORVA) of 7 to 10 years were similar between the HPV-positive and -negative (8.3%, 10.1%) samples, as were those above the recommended vaccination age of 45 and older (25.0%, 25.1%, respectively). Similar proportions of HPV-positive and -negative samples within the recommended vaccination age (RVA) were observed (50.0% and 42.0%, respectively), as were those within the vaccination catch-up age range of 27 to 45 years (16.7% and 21.8%, respectively).

A graphical analysis of the vaccination ages for the HPV-positive and HPV-negative samples was performed (Figure 2). This analysis demonstrated that the proportion of samples outside the recommended vaccination age between seven and ten years of age was similar between the HPV-positive (8.3%) and HPV-negative (10.1%) samples. Similarly, those within the recommended vaccination age between 11 and 26 years of age were also similar among the HPV-positive and -negative samples (50.0% and 42.0%, respectively), as were those within the vaccination catch-up age range of 27 to 45 years (16.7% and 21.8%, respectively). Finally, nearly identical proportions of samples above the recommended vaccination age (45 years and over) were found between the HPV-positive and -negative samples (25.0% and 25.1%, respectively).

**Figure 3:**
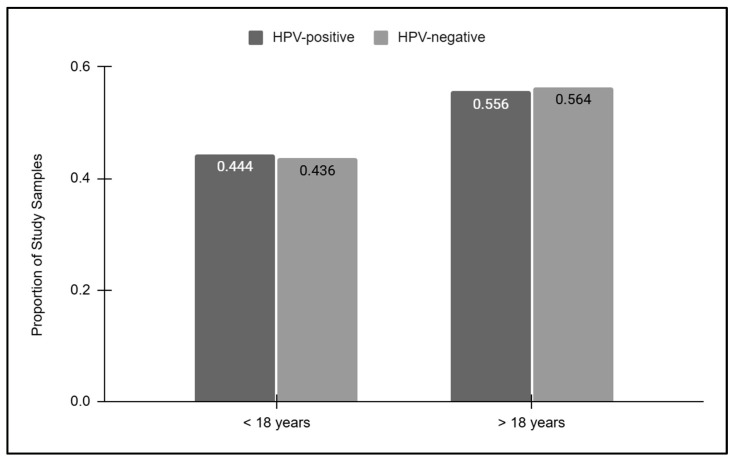
Pediatric and adult HPV screening results. Pediatric samples represented less than half of the HPV-positive (44.4%) and HPV-negative (43.6%) samples, while similar proportions of HPV-positive (55.6%) and HPV-negative (56.4%) samples were derived from adults, which was not statistically significant, *p* = 0.8399.

To more closely evaluate the relationship between age and HPV screening results, the HPV-positive and HPV-negative results were then sorted into pediatric and adult groups (Figure 3). These data demonstrated that slightly less than half of the HPV-positive (44.4%) and HPV-negative (43.6%) samples were derived from pediatric patients under the age of 18 years old. The proportion of HPV-positive (55.6%) and HPV-negative (56.4%) samples derived from adults over the age of 18 years old was also similar, which was not statistically significant, *p* = 0.8399.

### 3.4. Analysis of High-Risk Oral HPV Strains

**Figure 4:**
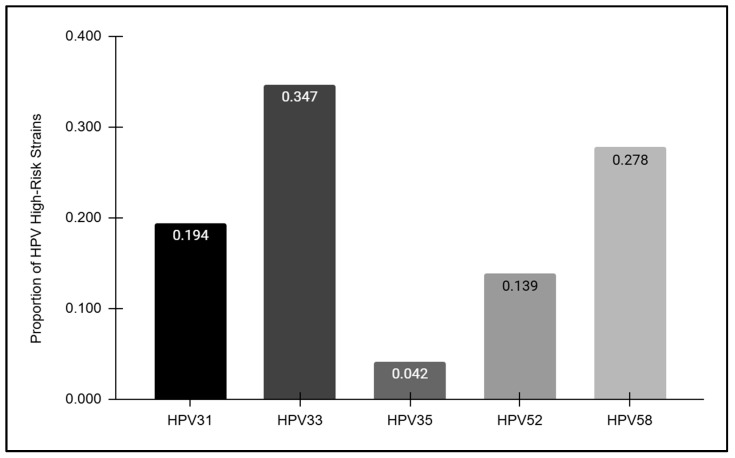
The prevalence of high-risk HPV strains. The highest proportion of HPV-positive samples was observed among HPV33 (34.7%), followed by HPV58 (27.8%), HPV31 (19.4%), HPV52 (13.9%), and HPV35 (4.2%).

The HPV-positive samples were further analyzed to determine the overall prevalence of each high-risk strain detected (Figure 4). These data revealed striking differences in prevalence among each of the high-risk strains analyzed, with the highest proportion observed among HPV33-positive samples (34.7%) followed by HPV58 (27.8%), HPV31 (19.4%), and HPV52 (13.9%). However, HPV35 was only observed among a very small proportion of the overall HPV-positive samples (4.2%).

### 3.5. Analysis of Pediatric HPV Vaccination Status

**Figure 5:**
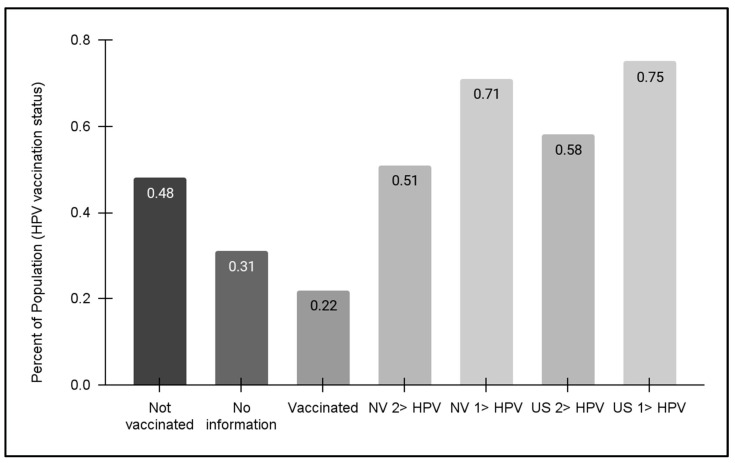
HPV vaccination status for pediatric patients. An analysis of data from pediatric patients aged 11–17 years revealed nearly half (48.0%) had not received any HPV vaccination. Less than one-quarter (22.1%) reported having received one or more doses of the HPV vaccine, while 30.9% of patients (or their parents and guardians) declined to offer this information. No data regarding HPV vaccination for the adult patient population were available at this time of this analysis. State and national HPV vaccination data revealed NV vaccination rates for HPV adolescents (13–17 years) were 51% (one dose) and 71% (two or more dosages), respectively, which were lower than the national averages of 58% and 75%, respectively.

Finally, vaccination status for the pediatric clinic population was evaluated (Figure 5). These data demonstrated that approximately half (48.0%) of pediatric patients within the clinic population self-reported that no HPV vaccine doses had been received. In addition, only 22.1% reported having received one or more doses of the HPV vaccine, although the clinic charting does not automatically separate and categorize the number of vaccinations (one, two, or three dosages). It is worth noting that 30.9% of patients (or their parents and guardians) declined to offer this information, which may suggest that HPV vaccination may be less likely among this subgroup.

Regional and national HPV vaccination status data from the Centers for Disease Control and Prevention (CDC) were also compiled and graphed for this analysis. These data demonstrated that state-level population statistics for at least one HPV vaccination dosage for adolescents (13–17 years) were approximately 71%, which was lower but similar to the national average of 75%. For the full dosage series of two or three HPV vaccinations, the percentages for Nevada and the US were lower (51% and 58%, respectively)—although both were much higher than the percentage of clinic patients within this same age range (13–17 years). Unfortunately, no data regarding HPV vaccination for adult patients were available at this time of this analysis.

## 4. Discussion

This study sought to determine the oral prevalence of high-risk HPV strains from within an existing salivary biorepository. These data revealed that several high-risk HPV strains, including most of those contained within the nine-valent HPV vaccine, were present. These data demonstrated that the oral prevalence for these high-risk HPV strains may be two-fold greater (40%) than another recent study of HPV16 and HPV18 prevalence (18.1%) from the same biorepository [36]. Moreover, these results correspond with other recent studies and reviews of oral HPV prevalence that screened for the additional non-HPV16 and non-HPV18 high-risk strains of HPV covered by the nine-valent HPV vaccine [37,38].

In addition, the longitudinal and temporal analysis incorporated into the study design demonstrated that these strains may be becoming more prevalent within this patient population over time—increasing from 23.1% to 40.0% between 2011 and 2019 [24,25,36]. These data reflect similar reviews and reports of increasing oral HPV prevalence that demonstrated marked increases in these high-risk HPV strains within the most recent ten to fifteen years among various patient populations [39,40,41]. An analysis of these data also revealed that the average prevalence of HPV over the first four years of this analysis (2011 to 2014) prior to the approval and introduction of the nine-valent vaccine in December 2014 was actually lower than the prevalence of these high-risk strains following this approval and vaccine rollout (2015 to 2019). It is important to note that these data also revealed that oral HPV positivity was equally distributed among both males and females, which has been a topic of increasing public health interest as HPV vaccination rates for young males within the recommended vaccination age continue to lag significantly behind those of young females [42,43,44].

These data revealed the overwhelming majority of samples with high-risk HPV came from patient samples within the recommended HPV vaccination age (11 to 26 years) or the recommended catch-up vaccination age range (27 to 45 years). These data correspond to other systematic reviews and meta-analyses that suggested oral HPV16 and HPV18 prevalence may also be found among patients within similar age ranges [45,46,47]. Despite numerous studies confirming the safety and efficacy of the nine-valent HPV vaccine, rising levels of vaccine hesitancy fueled by vaccine myths and misinformation spread on social media have created significant barriers and challenges to this straightforward and effective HPV-related prevention method [48,49,50,51]. Among the most effective strategies to increase provider willingness to engage and counter these misconceptions are data demonstrating the increasing trends and prevalence of HPV among vaccine-eligible adolescents and young adults, which may help to highlight the importance of these findings and the risks associated with failure to use evidence-based decisions among parents and caregivers [52,53,54,55].

This study also presents significant findings as the overwhelming majorities of both clinical biorepository samples and the clinical patient population are under-represented minorities [19,20,36]. These data are increasingly important as more and more studies have demonstrated that multiple challenges and barriers to vaccination in general, and HPV vaccination in particular, may be faced by ethnic and racial minorities within the United States, which have often led to lower rates of health prevention in general and HPV vaccination in particular that have been demonstrated by the rising prevalence of HPV among this patient population even after the introduction of the nine-valent HPV vaccine [56,57,58]. Despite the advances made in language proficiency and cultural competency, additional issues surrounding communication practices and long-standing trust issues between minority parents and physicians further complicate the landscape regarding HPV vaccination hesitancy [59,60,61,62].

These findings are also important considerations as more and more data now suggest that oral healthcare providers, including dentists and hygienists, may be able to engage more patients in a wide range of discussions that help to increase HPV awareness and HPV vaccination uptake [63,64]. For example, research from this group has demonstrated that vaccine myths, misconceptions, and hesitancy among dental students and post-graduate residents were mediated, in part, by up-to-date evidence-based knowledge regarding HPV vaccine safety and efficacy, as well as data and evidence regarding oral HPV prevalence and vaccine hesitancy within the clinical patient population [65,66]. This study adds to the growing body of evidence that may help to increase community awareness and engagement with HPV prevalence and the importance of HPV vaccination among vaccine-eligible adolescents and youth [67,68].

This study has several implicit limitations that should be considered. For example, this study does not provide the most up-to-date information on oral HPV prevalence due to the retrospective nature of this study. Therefore, any data regarding oral HPV prevalence between 2020 and 2024 were not available for analysis [19,20]. Another limitation is the nature of the convenience sample, which was drawn from a low minority-serving, low-income open clinic within a public dental school that may not accurately reflect the local or regional community at large [59,65,66]. Moreover, some limitations of this study included both the number and availability of previously collected samples, as well as any additional limitations and biases that could have been associated with the original sample collection that could not be controlled for within the parameters of the current study.

Despite these limitations, this study presented significant findings that may be important for public and oral health researchers for several reasons. First, these data revealed that the prevalence of high-risk oral HPV may be much higher than in other recent studies of healthy adult populations (4.5% to 6.6%) but within the range of oral HPV prevalence found by some oral health researchers (0.67% to 35%), which may be due to the extremely low vaccination rates among this low-income, minority patient population as well as the many challenges and barriers faced by these subgroups [41,69,70]. Next, these data demonstrated that despite the increase in vaccination rates among adolescents and teenagers in other industrialized countries following the nine-valent vaccine release, high oral HPV prevalence rates combined with low vaccination rates were observed within this patient population and may suggest that these phenomena could be present among other low-income, minority populations where prevention messages and implementation measures remain comparatively low and could be the basis for future studies in this area [71,72]. Finally, a more detailed analysis of these research studies also demonstrated nearly the same proportion of oral HPV-16 among males and females, which was similar to the result of this current study that showed no significant difference between high-risk oral HPV among females and males (47.2% and 52.8%, respectively) [71,72].

## 5. Conclusions

This study provides new evidence regarding the oral prevalence of the additional high-risk HPV strains covered by the nine-valent HPV vaccine evaluated over time. These data demonstrated that the oral HPV prevalence of these additional strains may be higher than that of HPV16 and HPV18 within this low-income and mostly minority patient population, which was found mainly among patients within the recommended or catch-up HPV vaccination age. Moreover, oral HPV prevalence also appears to be increasing over time, providing new evidence and support for the importance of the nine-valent HPV vaccine that covers these additional high-risk HPV strains, such as HPV31, HPV33, HPV52, and HPV58, and the public health measures that must be employed to increase knowledge, awareness, and acceptance.

## Figures and Tables

**Table 1:** Screening primer sequences.

Primer	Primer Sequence	Length (nt)	GC%	Tm
GAPDH forward	5′-ATCTTCCAGGAGCGAGATCC-3′	20 nt	55%	66 °C
GAPDH reverse	5′-ACCACTGACACGTTGGCAGT-3′	20 nt	55%	70 °C
HPV31 forward	5′-ATTCCACAACATAGGAGGAAGGTG-3′	24 nt	46%	66 °C
HPV31 reverse	5′-CACTTGGGTTTCAGTACGAGGTCT-3′	24 nt	50%	68 °C
HPV33 forward	5′-ATATTTCGGGGTCGTTGGGCA-3′	21 nt	52%	69 °C
HPV33 reverse	5′-ACGTCACAGTGCAGTTTCTCTACGT-3′	25 nt	48%	70 °C
HPV35 forward	5′-TCGGTGTATGTCTGTTGGAAAC-3′	22 nt	45%	65 °C
HPV35 reverse	5′-CATAGTCTTGCAATGTAGTTATTTCTCCA-3′	29 nt	34%	64 °C
HPV52 forward	5′-AAAGCAAAAATTGGTGGACGA-3′	21 nt	38%	63 °C
HPV52 reverse	5′-TGCCAGCAATTAGCGCATT-3′	19 nt	47%	66 °C
HPV52 forward	5′-GGCATGTGGATTTAAACAAAAGGT-3′	24 nt	38%	64 °C
HPV52 reverse	5′-TCTCATGGCGTTGTTACAGGTTAC-3′	24 nt	46%	67 °C

Key: nucleotide = nt; melting temperature = Tm.

**Table 2:** Demographic characteristics of study sample.

	Study Sample	Clinic Population	Statistical Analysis
Sex			
Female	49.4% (*n* = 124/251)	49.1%	X^2^ = 0.040, d.f. = 1 *p* = 0.8414
Male	50.6% (*n* = 127/251)	50.9%	
Race or ethnicity			
White or Caucasian	25.8% (*n* = 62/240)	29.4%	X^2^ = 0.4027, d.f. = 1 *p* = 0.5085
Minority	74.2% (*n* = 178/240)	70.6%	
Hispanic or Latino	30.4% (*n* = 73/240)	49.3%	
Black or African American	20.4% (*n* = 49/240)	10.7%	
Asian or Pacific Islander	20.0% (*n* = 48/240)	8.1%	
Other/Mixed race	3.3% (*n* = 8/240)	2.5%	
Age			
Average Range	25.19 years 7–51 years	26.35 0–89 years	Two-tailed *t*-test *p* = 0.1806

**Table 3:** Qualitative and quantitative analysis of study sample DNA.

Study Sample	DNA Concentration [ng/uL]	DNA Purity Ratio [A260:A280]
Pediatric samples (*n* = 110)	312.4 ng/uL +/− 63 range: 221–452 ng/uL	1.76 range: 1.71–1.82
Adult samples (*n* = 141)	431.2 ng/uL +/− 71 range: 347–558 ng/uL	1.79 range: 1.73–1.88

**Table 4:** Demographic analysis of HPV screening samples.

	HPV-Positive	HPV-Negative	Statistical Analysis
Sex			
Female	47.2% (*n* = 34/72)	50.3% (*n* = 90/179)	X^2^ = 0.360, d.f. = 1 *p* = 0.5485
Male	52.8% (*n* = 38/72)	49.7% (*n* = 89/179)	
Race or ethnicity			
White or Caucasian	20.8% (*n* = 15/72)	26.3% (*n* = 47/179)	X^2^ = 3.269, d.f. = 2 *p* = 0.1951
Minority	72.3% (*n* = 52/72)	70.4% (*n* = 126/179)	
Hispanic or Latino	29.2% (*n* = 21/72)	29.1% (*n* = 52/179)	
Black or African American	20.8% (*n* = 15/72)	18.9% (*n* = 34/179)	
Asian or Pacific Islander	18.1% (*n* = 13/72)	19.6% (*n* = 35/179)	
Not declared	11.1% (*n* = 8/72)	6.1% (*n* = 11/179)	
Age			
Average 7–10 years (ORVA) * 11–26 years (RVA) 26–45 years (VCA) 45 and older (ORVA) *	22.8 years 8.3% (*n* = 6/72) 50.0% (*n* = 36/72) 16.7% (*n* = 12/72) 25.0% (*n* = 18/72)	27.7 years 10.1% (*n* = 18/179) 43.0% (*n* = 77/179) 21.8% (*n* = 39/179) 25.1% (*n* = 45/179)	X^2^ = 2.676 d.f. = 3 *p* = 0.4443

Key: ORVA = outside recommended vaccination age, RVA = recommended vaccination age, VCA = vaccination catch-up age, * denotes outside recommended vaccination age (ORVA).

## Data Availability

The primary data may be available upon request from the corresponding author. These data are not publicly available according to the protection parameters for the study protocol, which were required by the IRB and OPRS for the study approval.
